# Early versus delayed treatment with glatiramer acetate: Analysis of up to 27 years of continuous follow-up in a US open-label extension study

**DOI:** 10.1177/13524585221094239

**Published:** 2022-06-29

**Authors:** Corey C Ford, Jeffrey A Cohen, Andrew D Goodman, John W Lindsey, Robert P Lisak, Christopher Luzzio, Amy Pruitt, John Rose, Horea Rus, Jerry S Wolinsky, Shaul E Kadosh, Emily Bernstein-Hanlon, Yafit Stark, Jessica K Alexander

**Affiliations:** Department of Neurology, University of New Mexico Health Sciences Center, The University of New Mexico, Albuquerque, NM, USA; Mellen Center for Multiple Sclerosis, Neurological Institute, Cleveland Clinic Foundation, Cleveland, OH, USA; Department of Neurology, University of Rochester, Rochester, NY, USA; Department of Neurology, University of Texas Health Science Center at Houston (UTHouston), Houston, TX, USA; Department of Neurology and Department of Biochemistry, Microbiology and Immunology, School of Medicine, Wayne State University, Detroit, MI, USA; Departments of Neurology and Engineering, University of Wisconsin–Madison, Madison, WI, USA; Department of Neurology, University of Pennsylvania Medical Center, Philadelphia, PA, USA; Imaging and Neuroscience Center, School of Medicine, The University of Utah, Salt Lake City, UT, USA; Department of Neurology, School of Medicine, University of Maryland, Baltimore, MD, USA; Department of Neurology, University of Texas Health Science Center at Houston (UTHouston), Houston, TX, USA; Innovative Research and Development, Teva Pharmaceuticals, Netanya, Israel; Global Clinical Programming, Teva Pharmaceuticals, Netanya, Israel; Global Clinical Development, Teva Pharmaceuticals, Netanya, Israel; Global Medical Affairs, Teva Pharmaceuticals, West Chester, PA, USA/Jazz Pharmaceuticals, Palo Alto, CA, USA

**Keywords:** Clinical trial, disease-modifying therapies, glatiramer acetate, multiple sclerosis, quality of life, relapsing/remitting

## Abstract

**Background:**

Glatiramer acetate (GA) is US-approved for relapsing multiple sclerosis.

**Objectives:**

To describe GA long-term clinical profile. To compare effectiveness of early start (ES) versus delayed start (DS; up to 3 years) with GA.

**Methods:**

Phase 3 trial participants entered a randomized placebo-controlled period then an open-label extension (OLE) with GA.

**Results:**

Overall, 208 out of 251 (82.9%) randomized participants entered the OLE; 24 out of 101 (23.8%, ES) and 28 out of 107 (26.2%, DS) participants completed the OLE. Median GA treatment was 9.8 (0.1–26.3) years. Annualized change in Expanded Disability Status Scale (EDSS) score was lower with ES versus DS (*p* = 0.0858: full study; *p* = 0.002; Year 5). Participants with improved/stable EDSS was consistently higher with ES versus DS: 40.3% versus 31.6% (*p* = 0.1590; full study); 70.8% versus 55.6% (*p* = 0.015; Year 5). ES prolonged time-to-6-month confirmed disease worsening (CDW) versus DS (9.8 vs 6.7 years), time-to-12-month CDW (18.9 vs 11.6 years), and significantly reduced time-to-second-6-month CDW (*p* = 0.0441). No new safety concerns arose.

**Conclusion:**

GA long-term treatment maintained clinical benefit with a similar safety profile to phase 3 results; a key limitation was that only 25% of participants completed the OLE. Early initiation of GA had sustained benefits versus delayed treatment.

## Introduction

Multiple sclerosis (MS) is a chronic disabling disease usually commencing in early adulthood.^1^ Relapsing MS (RMS) treatment recommendations encourage early intervention with disease-modifying therapies (DMTs) to optimize long-term clinical outcomes.^2–4^ Early treatment initiation and the chronic and progressive nature of MS means patients may remain on DMTs for decades.^5^ Patients with MS switch DMTs due to incomplete disease control and/or intolerability^5,6^; ~30%^7^–60%^8^ of patients discontinue their first DMT. Consequently, DMT long-term safety and efficacy data are limited.

Glatiramer acetate (GA) was FDA-approved for RMS in 1996 (20 mg/mL subcutaneously once-daily (QD)),^9^ and 2014 (40 mg/mL subcutaneously three times weekly (TIW)),^10,11^ and is licensed in 59 countries.^12–14^ In the phase 3 trial, the GA-treated group had a 29% reduction in relapse rate over 2 years (primary endpoint) versus placebo.^9^ In the double-blind extension of up to 11 months, relapse rate was reduced by 32% versus placebo and clinical worsening (change in the Expanded Disability Status Scale (EDSS) score) was significantly lower with GA (21.6% vs 41.6% (placebo)) at 35 months.^15^ Most participants entered a long-term, open-label extension (OLE) study. This extension demonstrated sustained GA efficacy in RMS for 6,^16,17^ 10,^18^ and 15^19^ years. GA was well tolerated and demonstrated a sustained, favorable safety profile.^9,15–19^

The objective of this paper is to describe the long-term clinical profile of GA up to 27 years in participants with RMS as part of the final OLE analyses, and for the first time, to compare the effectiveness of an early start (ES) of GA treatment versus an up to 3-year delayed start (DS).

## Methods

### Study design

All participants in the randomized placebo-controlled 24-month study were eligible for the double-blind extension study. Participants from that study were eligible for the OLE. All OLE participants received GA at 20 mg/mL QD, with an option to switch to 40 mg/mL TIW when available.

Eligibility criteria and study procedures have been described.^9,15–19^ Any participant who stopped GA or took another DMT was withdrawn from the OLE. All participants provided written informed consent for each study part. The 11 original US academic centers participated in the OLE and their institutional review boards periodically reviewed and approved each site’s ongoing participation.

### Outcomes

EDSS scores were assessed every 6 months during the first 13 years of the OLE, then every 12 months. Key endpoints included annualized change in EDSS score, time-to-EDSS score of 4, 6, and 8, and proportion of participants with a stable/improved EDSS score (⩽0.5-point increase from baseline). Annualized changes in ambulatory index^20^ and functional systems score (FSS) pyramidal function^21^ were monitored.

Six-month confirmed disease worsening (CDW) and 12-month CDW were defined as an increase in EDSS score of ⩾ 1 point from baseline (baseline EDSS score ⩽ 5.0) or an increase of ⩾ 0.5 points from baseline (baseline EDSS score ⩾ 5.5), confirmed after at least 6 or 12 months, respectively. Disease worsening could not be assessed during a relapse. Endpoints included time-to-6-month CDW, time-to-12-month CDW and time-to-second-6-month CDW; and proportion of participants free from these endpoints. Analysis of time-to-second-6-month CDW included all EDSS measurements taken following onset of the first 6-month CDW, censoring participants who were free from either first or second 6-month CDW. The proportion of participants who were disease-activity free (no evidence of disease activity (NEDA-2), that is, no clinical evidence of relapse activity or disability worsening) was assessed. Participants meeting NEDA-2 criteria were required to have no confirmed relapse and no confirmed worsening of EDSS score during the study.

Relapse was defined as the appearance/reappearance of one or more neurologic abnormalities persisting for at least 48 hours, preceded by a stable or improving neurological state of at least 30 days. An event was counted as a relapse only when symptoms were accompanied by observed objective neurological changes, including a ⩾ 0.5-point increase in EDSS score^21^ versus the previous evaluation. Annualized relapse rate (ARR) and proportion of relapse-free participants were assessed.

Safety endpoints were monitored throughout, including treatment emergent adverse events (TEAEs, including hepatic effects) leading to study discontinuation, serious TEAEs, immediate post-injection reactions (IPIRs), and injection-site reactions (ISRs).

### Statistical analyses

Annualized change outcomes were analyzed using mixed models for repeated measurements. Time-to-event outcomes were displayed using Kaplan–Meier survival distribution curves and analyzed using Cox proportional hazards models. Proportions of participants were analyzed using logistic regression models. ARRs were evaluated using exposure-weighted negative binomial regression models. All effectiveness analyses were adjusted for baseline EDSS score and number of relapses (log number when applicable) in the 2 years prior to study initiation. Annualized TEAEs, IPIRs, and ISRs were compared between cohorts using exposure-weighted negative binomial regression. No multiplicity adjustment was performed. Statistical results are presented as model-adjusted estimates.

## Results

### Participant disposition, demographics, and GA exposure

Of the 251 participants randomized to GA or placebo (original study and double-blind extension), 208 (82.9%) entered the OLE; ES: *n* = 101; DS: *n* = 107. Fifty-two (25%) participants completed the OLE (ES: *n* = 24 (23.8%) DS: *n* = 28 (26.2%); Supplemental Figure 1). Overall, 199 (79.3%) participants discontinued (ES: *n* = 101/125; DS: *n* = 98/126), mainly due to participant withdrawal (115/199; no classification for lack of efficacy was considered at study design). Overall, 39 out of 208 (18.8%) OLE participants switched to GA 40 mg/mL TIW, and 1 out of 39 (2.6%) switched back to 20 mg/mL QD.

Baseline demographic and clinical characteristics at randomization were comparable between groups (Table 1).

**Table 1.** Participant demographics and disease characteristics at randomization.

Parameter Mean (SD) unless stated otherwise	Early start (*n* = 125)	Delayed start (*n* = 126)	All (*N* = 251)
Demographics			
Age at randomization, years	34.6 (6.0)	34.3 (6.5)	34.4 (6.2)
Age at termination, years	48.5 (11.1)	48.6 (11.3)	48.6 (11.2)
Sex, female, *n* (%)	88 (70)	96 (76)	184 (73)
Baseline disease characteristics			
Age at onset of first MS symptoms, years	27.3 (5.9)	27.6 (6.5)	27.5 (6.2)
Duration of disease at randomization, years	7.3 (4.9)	6.6 (5.1)	7.0 (5.0)
EDSS score at randomization	2.8 (1.2)	2.4 (1.3)	2.6 (1.3)
Number of relapses during the last 2 years at randomization	2.9 (1.3)	2.9 (1.1)	2.9 (1.2)

SD: standard deviation; MS: multiple sclerosis; EDSS: Expanded Disability Status Scale.

Supplemental Figures 2 and 3 depict duration of follow-up and distribution of participants by duration of GA exposure from randomization. Median (range) time in the study was 11.4 (0.1–26.9) years (ES: 11.2 (0.3–26.9); DS: 11.7 (0.1–26.3)). Median (range) duration of GA treatment was 9.8 (0.1–26.3) years (ES: 9.4 (0.1–26.3); DS: 10.6 (0.1–23.5)). Overall, 74 out of 251 (29.5%) randomized participants had > 20 years GA exposure.

### Disability outcomes

**Figure 1.**
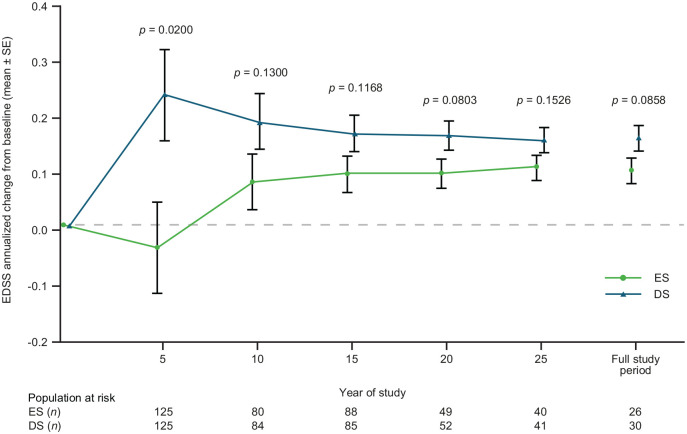
Annualized change from baseline in EDSS score by treatment group. The *p* values are from the mixed model repeated measures. EDSS: Expanded Disability Status Scale; ES: early start; DS: delayed start; SE: standard error.

At randomization, mean (SD) EDSS scores were 2.8 (1.2; ES) and 2.4 (1.3; DS), which increased to 3.29 (2.13) and 3.98 (2.58), respectively at OLE completion. Change in EDSS score over the full study was numerically lower for the ES versus DS group (Figure 1). Annualized change from baseline in EDSS score at Year 5 was significantly lower in the ES versus DS group: mean difference (95% confidence interval (CI)): −0.278 (−0.511; −0.044); *p* = 0.020.

**Figure 2.**
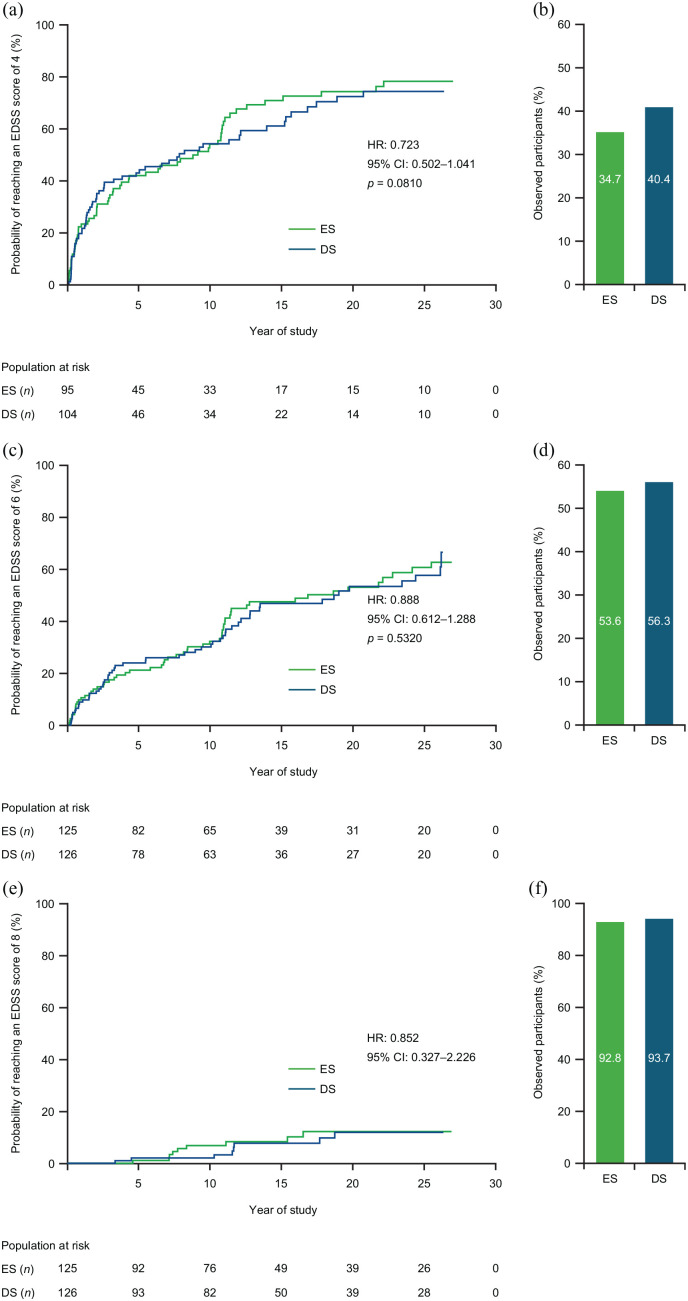
Time-to-EDSS score and proportion of participants not reaching an EDSS score of (a and b) 4, (c and d) 6, or (e and f) 8. Kaplan–Meier curves. HR, CI, and *p* values are from the Cox proportional hazards model. CI: confidence interval; EDSS: Expanded Disability Status Scale; ES: early start; DS: delayed start; HR: hazard ratio.

No significant differences between ES and DS groups in time-to-EDSS score of 4 (Figure 2(a)), 6 (Figure 2(c)), or 8 (Figure 2(e)) over the full study were seen. Estimated median time (95% CI) to reach EDSS 4 was 9.09 (3.75–10.79; ES) and 7.79 (3.21–13.90; DS), and EDSS 6 was 16.80 (10.97–25.47; ES) and 18.98 (12.11–26.17; DS) years; median time-to-EDSS 8 was not calculated due to high-censoring rate. Proportions of participants not reaching defined EDSS thresholds were numerically lower in the ES group versus the DS group (Figures 2(b), (d) and (f)).

**Figure 3.**
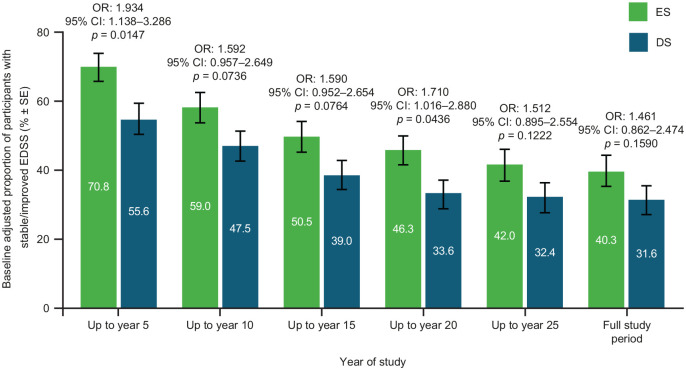
Proportion of participants with stable/improved EDSS by treatment group. Stable/improved EDSS scores were defined as up to a 0.5-point increase from baseline, worsened EDSS scores were defined as a > 0.5 increase from baseline. Bars display percentage estimates ±SEs. OR, 95% CI and *p* values are from logistic regression model results; covariates used were baseline EDSS score and log of the number of relapses in the 2 years prior to study. CI: confidence interval; EDSS: Expanded Disability Status Scale; ES: early start; DS: delayed start; OR: odds ratio.

Baseline-adjusted proportion of participants with stable/improved EDSS up to Year 5 (odds ratio: 1.934; 95% CI: 1.138–3.286; *p* = 0.0147) and up to Year 20 (odds ratio: 1.710; 95% CI: 1.016–2.880; *p* = 0.0436) was significantly higher in the ES versus DS group, and remained numerically higher throughout the OLE (Figure 3).

**Figure 4.**
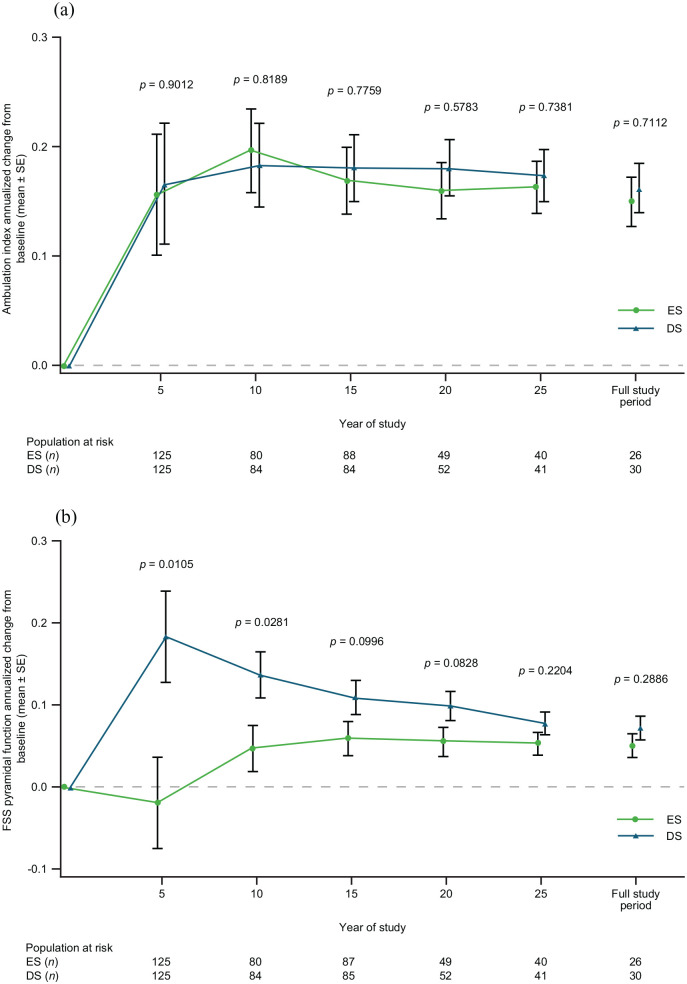
Annualized change from baseline in (a) ambulation index and in (b) FSS pyramidal function, by treatment group. The *p* values are from the mixed-model repeated measures. ES: early start; DS: delayed start; FSS: functional systems score; SE: standard error.

Annualized change from baseline in ambulation index was similar between the groups over the full study (Figure 4(a)). Annualized change from baseline in FSS pyramidal function was significantly lower at Year 5 and Year 10 in the ES versus DS group (Figure 4(b)).

**Figure 5.**
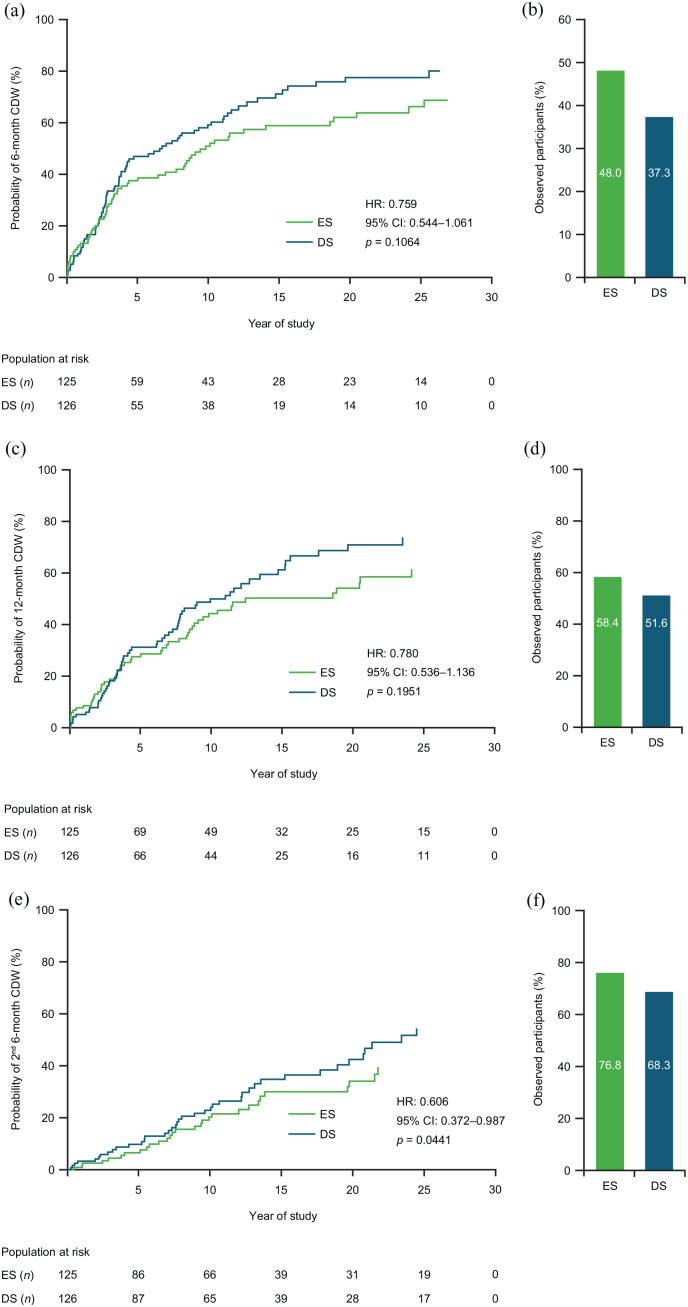
(a) Time-to-6-month CDW, (c) time-to-12-month CDW, and (e) time-to-second-6-month CDW; and participants free from (b) 6-month CDW, (d) 12-month CDW, and (f) 6-month CDW, by treatment group. Kaplan–Meier curves. HR, CI, and *p* values from the Cox proportional hazards model; covariates used were baseline EDSS score and log of the number of relapses in the 2 years prior to study. CDW defined as an increase in EDSS of 1 point from baseline for participants with a baseline EDSS of 5.0, or an increase in EDSS of 0.5 points from baseline for participants with a baseline EDSS of ⩾ 5.5. CDW: confirmed disease worsening; CI: confidence interval; EDSS: Expanded Disability Status Scale; ES: early start; DS: delayed start; HR: hazard ratio.

ES treatment prolonged the median time-to-6-month CDW (9.82 years) versus DS treatment (6.71 years; Figure 5(a)). This finding was confirmed when sex was included in the model (hazard ratio (HR): 0.760; 95% CI: 0.543–1.065; *p* = 0.1108), suggesting no differences between sexes. Observed proportions of participants remaining free from 6-month CDW were 48.0% (ES) and 37.3% (DS; Figure 5(b)). Median time-to-12-month CDW was 18.9 years (ES) and 11.6 years (DS; Figure 5(c)). The observed proportion of participants who remained free from 12-month CDW was 58.4% (ES) and 51.6% (DS; Figure 5(d)). Time-to-second-6-month CDW was significantly reduced by ES treatment versus DS treatment (Figure 5(e)). The observed proportion of participants who remained free from a second 6-month CDW was 76.8% (ES) and 68.3% (DS; Figure 5(f)).

**Figure 6.**
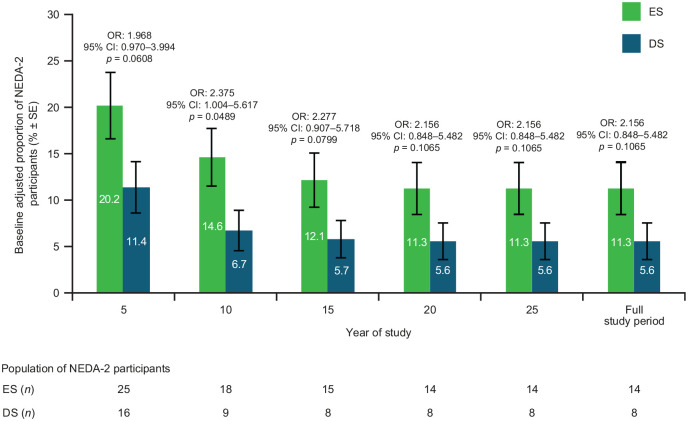
Proportion of participants meeting NEDA-2 criteria by treatment group. NEDA was defined as no relapse and no confirmed disease worsening. Bars display percentage estimates ±SEs. OR, 95% CI, and *p* values are from logistic regression model results; covariates used were baseline EDSS score and log of the number of relapses in the 2 years prior to study. CI: confidence interval; ES: early start; DS: delayed start; NEDA: no evidence of disease activity; OR: odds ratio; SE: standard error.

Baseline-adjusted proportion of disease-activity free participant criteria over the full study was 11.3% for ES and 5.6% for DS (Figure 6).

### Relapse outcomes

**Figure 7.**
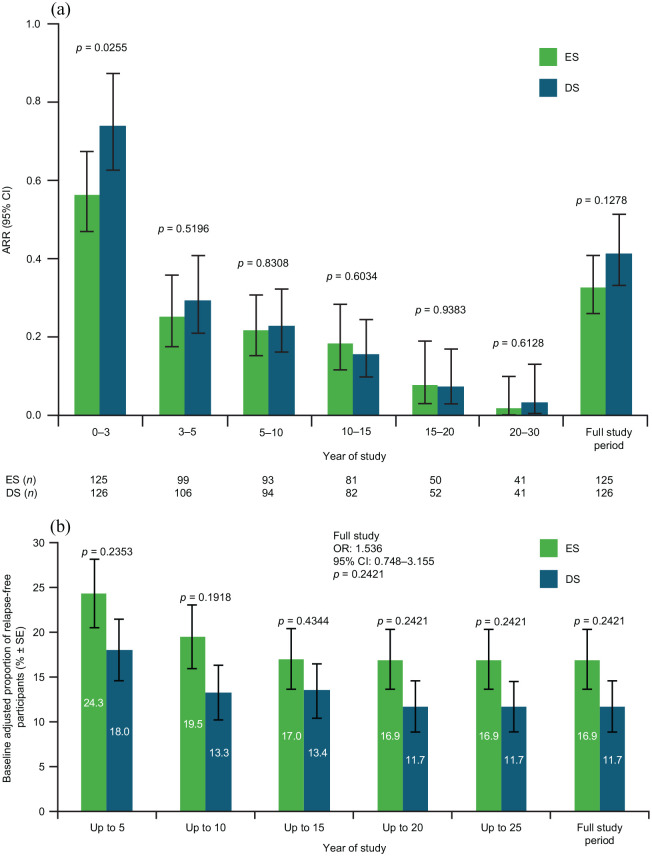
(a) ARR and (b) proportion of relapse-free participants by treatment group. The *p* values are from the negative binomial regression model (a) and logistic regression model (b). ARR: annualized relapse rate; CI: confidence interval; ES: early start; DS: delayed start; OR: odds ratio; SE: standard error.

Overall, ARR did not differ between ES (0.328) and DS participants (0.414; risk ratio (RR): 0.792; 95% CI: 0.586–1.069; *p* = 0.1278; Figure 7(a)). Accumulated ARR over Years 0–3 was significantly lower for ES versus DS participants (RR: 0.7613; 95% CI: 0.599–0.967; *p* = 0.0255). Baseline-adjusted proportion of participants without relapse over the full study was 16.9% for ES and 11.7% for DS (Figure 7(b)).

### Safety

Overall, 38 out of 232 (16.4%) participants had at least one TEAE leading to discontinuation; most common TEAEs leading to study discontinuation were injection-site erythema and injection-site pain (Table 2). Eighty-seven participants (37.5%) had at least one serious TEAE; the most common being urinary tract infection, chest pain, and dehydration (Table 2). Overall, 23.7% of participants (*n* = 55) had at least one IPIR, most commonly chest pain and vasodilatation (flushing). Most participants, 87.1% (*n* = 202) had at least one ISR, most commonly erythema and pain (57.3%).

**Table 2.** Frequency and incidence of treatment-emergent adverse events (incidence ⩾ 2%), immediate post-injection reactions (incidence ⩾ 2%), and injection-site reactions (incidence ⩾ 5%).

TEAE MedDRA preferred term	GA (*N* and *PY*)	
	*N* = 232, *PY* = 3088.2	
	Events, *n*	Participants, *n* (%)
Participants with at least one TEAE leading to study discontinuation	101	38 (16.4)
Injection-site erythema	8	8 (3.4)
Injection-site pain	7	6 (2.6)
Injection-site swelling	5	5 (2.2)
Pregnancy	8	5 (2.2)
Participants with at least one serious TEAE	329	87 (37.5)
Urinary tract infection	11	9 (3.9)
Chest pain	10	7 (3.0)
Dehydration	9	7 (3.0)
Uterine leiomyoma	7	6 (2.6)
Back pain	6	5 (2.2)
Depression	10	5 (2.2)
IPIR combination of categories	*N* = 232, *PY* = 2845.9	
	Cases, *n*	Participants, *n* (%)
Participants with ⩾ 1 IPIR	174	55 (23.7)
Chest pain, vasodilatation (flushing)	23	16 (6.9)
Chest pain, dyspnea, vasodilatation (flushing)	20	14 (6.0)
Dyspnea, vasodilatation (flushing)	18	13 (5.6)
Chest pain, dyspnea	9	7 (3.0)
Palpitations, vasodilatation (flushing)	9	7 (3.0)
ISR MedDRA preferred term	*N* = 232, *PY* = 2845.9	
	Events, *n*	Participants, *n* (%)
Participants with ⩾ 1 ISR	2059	202 (87.1)
Erythema	352	152 (65.5)
Pain	315	133 (57.3)
Mass	370	81 (34.9)
Pruritis	265	75 (32.3)
Swelling	202	69 (29.7)
Hemorrhage	73	30 (12.9)
Induration	77	45 (19.4)
Urticaria	76	37 (15.9)
Reaction	28	19 (8.2)
Inflammation	23	14 (6.0)
Warmth	23	21 (9.1)
Bruising	68	34 (14.7)

GA: glatiramer acetate; PY: patient-years of study; TEAE: treatment-emergent adverse event; MedDRA: Medical Dictionary for Regulatory Activities; IPIR: immediate post-injection reaction; ISR: injection-site reaction. TEAEs leading to study discontinuation were those classified in “action taken with the study treatment” attribute as “drug withdrawn.” IPIRs were defined as at least two or more symptoms occurring immediately after injection that include the following symptoms: vasodilatation (flushing), chest pain, palpitations, anxiety, dyspnea, constriction of the throat (laryngospasm), and urticaria.

Mean annualized TEAEs were higher in the ES group versus the DS group over Years 0–3 (RR: 1.214; 95% CI: 1.025–1.438; *p* = 0.0250), and were non-significant for the remaining period. This pattern was observed over Years 0–3 for IPIRs (RR: 3.988; 95% CI: 1.845–8.621; *p* = 0.0004) and ISRs (RR: 2.418; 95% CI: 1.818–3.216; *p* < 0.001). Mean annualized IPIRs were similar between cohorts for the remaining period, whereas mean annualized ISRs were higher for the DS versus ES group for Years 3–5 (RR: 0.459; 95% CI: 0.209–1.008; *p* = 0.0525) and 5–10 (RR: 0.366; 95% CI: 0.135–0.990; *p* = 0.0476) and similar between cohorts for the remaining period.

## Discussion

This OLE of the GA pivotal trial was completed 27 years after study initiation. The present analyses extend the findings reported at 6,^16^ 10,^18^ and 15^19^ years follow-up. For the first time, ES versus DS analyses were conducted on this integrated dataset. Several analyses demonstrated a sustained benefit up to 27 years of GA treatment started early versus a 3-year delay in initiation. Significant differences between ES versus DS treatment were seen up to Year 5; that is, significantly lower annualized change in EDSS score from baseline, a higher proportion of participants with improved/stable EDSS score, a lower incidence of a second 6-month CDW, and a lower annualized change in FSS pyramidal function from baseline; numerical differences were seen in most other disability endpoints. ES GA treatment significantly reduced accumulated ARR over Years 0–3 versus DS treatment. Safety findings were consistent with the original phase 3 trial.

Current analyses of this OLE focused on disability and disease worsening parameters, as these endpoints are clinically relevant in the long-term MS course. RMS transition to secondary progressive MS (SPMS) includes progressive worsening of neurologic function (disability accumulation) and relapses become less frequent over time.^22–24^ Furthermore, the participants in this OLE were considered to have very active RMS at initiation with factors associated with high risk of disability and progression.^25^

EDSS score^21^ was assessed at the start of the US pivotal trial^9^ and used throughout both extensions.^15–19^ Mean EDSS scores did increase with time indicating worsening disability.^21^ However, worsening was apparently slow as mean score at OLE completion at up to 27 years of GA treatment indicated that, on average, the participants were fully ambulatory without aid. Naturally, these results should be viewed in the context of potential participant withdrawal due to relapses or disease worsening.

Compared with DS GA treatment, ES GA treatment significantly decreased annualized change in EDSS score, increased the proportion of participants with improved or stable EDSS score, and decreased annualized change in FSS pyramidal function up to Year 5 of the OLE. At all other time points for these parameters, and all timepoints for other EDSS analyses, results with ES GA treatment were generally numerically better versus DS treatment; although, perhaps due to small participant numbers over time, these differences did not attain statistical significance. These findings are in keeping with other observational studies. The risk of reaching all disability outcomes was significantly lower in patients with MS treated within 1.2 years from onset versus those with a delayed treatment start.^26^ High-efficacy therapy started within 2 years of disease onset was also associated with less disability after 6–10 years versus those with a later treatment start.^27^

Participants in this OLE experienced worsening disabilities over time, as expected.^28^ At Year 15, in the ongoing GA group, the proportion of participants reaching EDSS scores of 4, 6, and 8 were 38%, 18%, and 3%, respectively,^19^ which were lower than in the present analysis, although a direct comparison is not possible as different analyses were conducted. Furthermore, proportion of participants with stable or improved EDSS score was 57% at Year 15,^19^ which is higher than the present data. Other disability parameters in this OLE also tended to indicate that ES GA treatment was better than DS GA treatment over time. It is unclear whether these differences are explained by a long-lasting effect of early treatment, the unfortunate downside of assignment to the initial placebo-treated group, a similar responsiveness to GA once initiated, or the emergence of a sub-cohort of responsive participants due to attrition from the open-label nature of the OLE.

DMT treatment of RMS reduces the risk of SPMS development, although such therapies do not impact disability accumulation in SPMS.^4,23,29^ Thus, evaluating disease worsening is relevant in long-term studies. However, confirming progression to SPMS is challenging.^30,31^ The present analyses showed that ES GA treatment numerically prolonged median time-to-6-month and -12 month CDW, and significantly reduced median time-to-second-6-month CDW versus the DS group. The unique long-term nature of this study enabled, to our knowledge, the first use of time-to-second-6-month CDW. Furthermore, the proportion of disease-activity-free participants was higher with ES GA versus DS GA. In the 15-year analyses, 35% of participants treated with GA developed SPMS (an increase >1.0 in EDSS score sustained for 12 months, without relapses occurring during that period).^19^ In the present 25-year analysis, 41.4% (ES) and 48.4% (DS) of participants met this definition. This progression was also apparently slow, although interpretation is difficult without a placebo control.

ARR decreased with time over the full study period in both ES and DS GA groups, supporting the focus of the present analyses on disability parameters as being more relevant. This reduction in relapses is as expected, given that many patients with RMS progress within 15–30 years with fewer relapses.^22–24^ However, the results also likely reflect continued GA efficacy in RMS in the long-term, as these findings extend previous observations GA treatment reducing relapses in RMS,^11–13^ and in interim analyses of this trial.^9,15–19^ The highest ARR was observed in Years 0–3 of the OLE, with participants in the ES group having significantly fewer relapses versus the DS group. Subsequently, ARR was numerically lower in the ES group versus the DS group. Furthermore, the proportion of relapse-free participants was numerically higher in the ES GA group versus the DS group throughout, although this parameter did gradually decline.

Continuous use of GA over 25 years was generally well tolerated. The AE profile was comparable with safety findings reported throughout this trial.^9,15–19^ The most common AEs were ISRs, which are well known with GA.^10–13^ These findings demonstrate that participants were willing to self-inject with GA either QD or TIW throughout the 27-year period. Idiosyncratic and possibly immune-mediated dose-independent hepatocellular injury with an unpredictable latency ranging from days to years have recently been described following treatment with GA^11,32^; however, no new safety signals were identified during the OLE study.

GA and interferons were approved for RMS treatment in the mid-1990s, and many more DMTs have been approved since,^33^ especially in the last 5 years. Thus, long-term follow-up data for recent DMTs are limited. However, long-term follow-up data on long-established DMTs in RMS are also limited, as such evaluations are usually retrospective, with infrequent patient assessments, and incomplete knowledge on other DMT treatments over time.^34,35^ Importantly, with prospectively collected data for more than 25 years, this GA OLE dataset is unique and is the longest clinical study to routinely and continuously evaluate the safety and effectiveness of any DMT in participants with RMS, and the results reinforce GA benefits by contemporary standards. Collectively, disability parameters and relapse data in this OLE analysis of over 25 years of continuous GA treatment highlight the sustained clinical benefits of GA, particularly with initiating early treatment, in keeping with recommendations for early intervention with DMTs in RMS to optimize outcomes.^2–4^

A key limitation of the OLE analyses is that only 25% of participants overall (23.8% ES; 26.2% DS) completed the study. Thus, there is a potential attrition bias caused by participant withdrawals. However, the reasons for withdrawal were generally similar in both groups, except for death (1% vs 3%), AEs (5% vs 10%), and other reasons (16% vs 10%). In addition, selection of several endpoints was based on pragmatic considerations, for example, NEDA-2 was used instead of NEDA-3 as magnetic resonance imaging data were not collected; and when the original study began, ambulation index was included and not the more recent 25-feet timed walk.

## Conclusion

This OLE represents the longest prospective study of continuous disease-modifying monotherapy in RMS, with > 25 years of GA monotherapy treatment experience in a single cohort. Based on this unique prospective dataset on GA, long-term (and short-term up to 5 years) GA monotherapy demonstrated clinical benefits in RMS based on worsening of disability and relapses. GA safety profile in the original phase 3 part of this study was maintained for over 25 years in the OLE. These findings also suggest that earlier initiation of GA treatment, versus DS, has overall sustained clinical benefits in RMS treatment.

## Supplemental Material

sj-docx-1-msj-10.1177_13524585221094239 – Supplemental material for Early versus delayed treatment with glatiramer acetate: Analysis of up to 27 years of continuous follow-up in a US open-label extension study Click here for additional data file. Supplemental material, sj-docx-1-msj-10.1177_13524585221094239 for Early versus delayed treatment with glatiramer acetate: Analysis of up to 27 years of continuous follow-up in a US open-label extension study by Corey C Ford, Jeffrey A Cohen, Andrew D Goodman, John W Lindsey, Robert P Lisak, Christopher Luzzio, Amy Pruitt, John Rose, Horea Rus, Jerry S Wolinsky, Shaul E Kadosh, Emily Bernstein-Hanlon, Yafit Stark and Jessica K Alexander in Multiple Sclerosis Journal
