# Supplementary material for: Early versus delayed treatment with glatiramer acetate: Analysis of up to 27 years of continuous follow-up in a US open-label extension study
Source: Mult Scler. 2022 Jun 29;28(11):1729–43. doi: 10.1177/13524585221094239 (PMC9442630; doi:10.1177/13524585221094239)
Supplement: sj-docx-1-msj-10.1177_13524585221094239 – Supplemental material for Early versus delayed treatment with glatiramer acetate: Analysis of up to 27 years of continuous follow-up in a US open-label extension study [file sj-docx-1-msj-10.1177_13524585221094239.docx]

**Supplemental Appendix**

**Supplemental Figure 1.** Participant disposition.


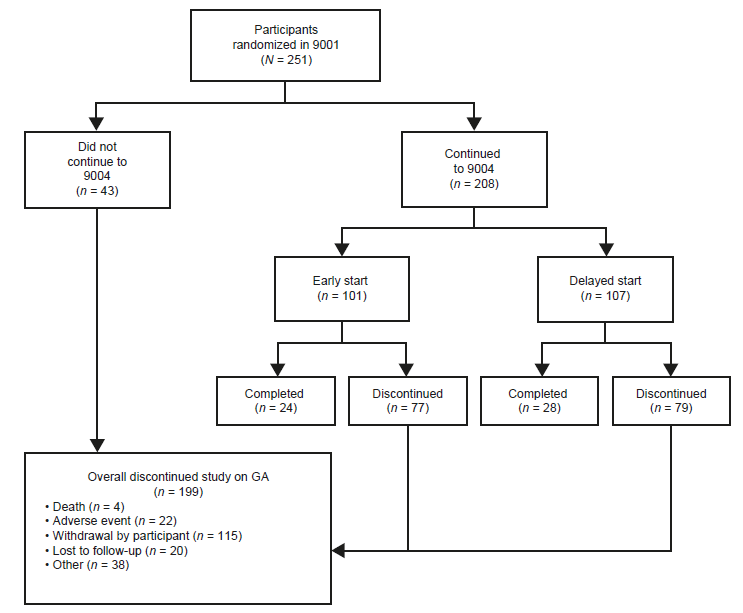


Specific reasons for discontinuation from study 9004, n (%) participants for ES vs DS groups: death 1 (<1) vs 3 (3); adverse event 5 (5) vs 11 (10); withdrawal by patient 48 (48) vs 45 (42); lost to follow-up 7 (7) vs 9 (8); other reasons 16 (16) vs 11 (10). Other reasons included: disease progression/treatment failure; patient did not keep/unable to attend scheduled appointments; continued secondary progression; patient decided to participate in a different clinical study; non-compliance; patient moved to alternative therapy; patient did not continue in the study; patient required other immunomodulating therapy.

GA: glatiramer acetate; 9001: the original double-blind study; 9004: open-label extension.

**Supplemental Figure 2.** K-M display of years to study discontinuation.


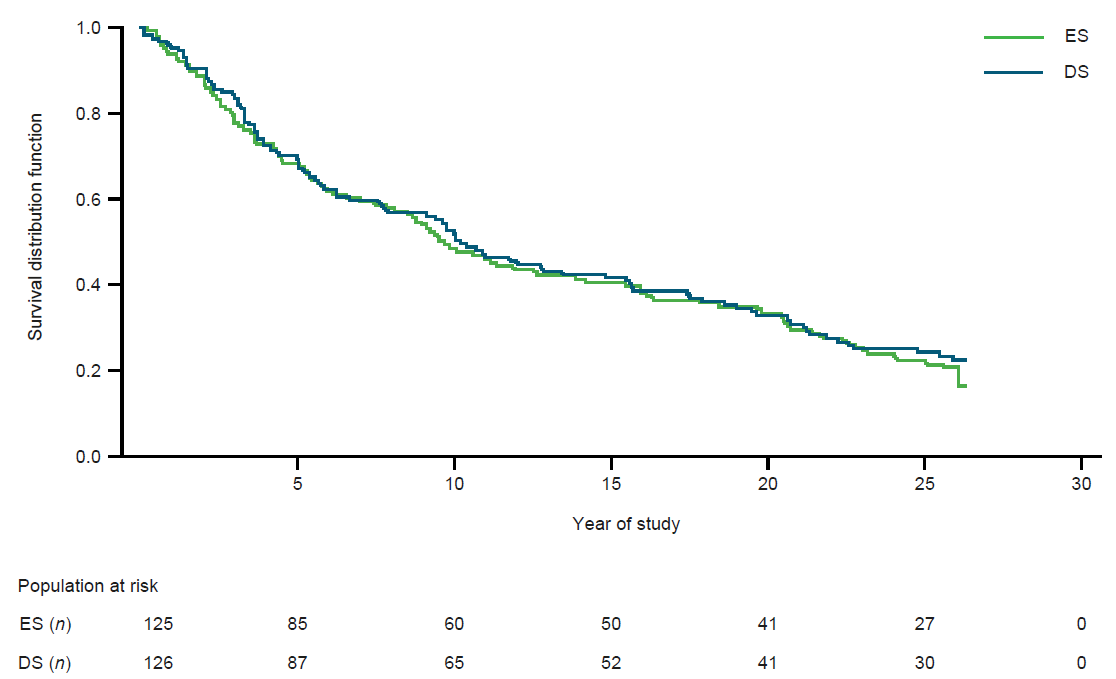


Kaplan-Meier curves. Participant duration in study was defined as the last observation in the study.

ES: early start; DS: delayed start; K-M: Kaplan-Meier.

**Supplemental Figure 3.** Distribution of participants by duration of GA exposure.


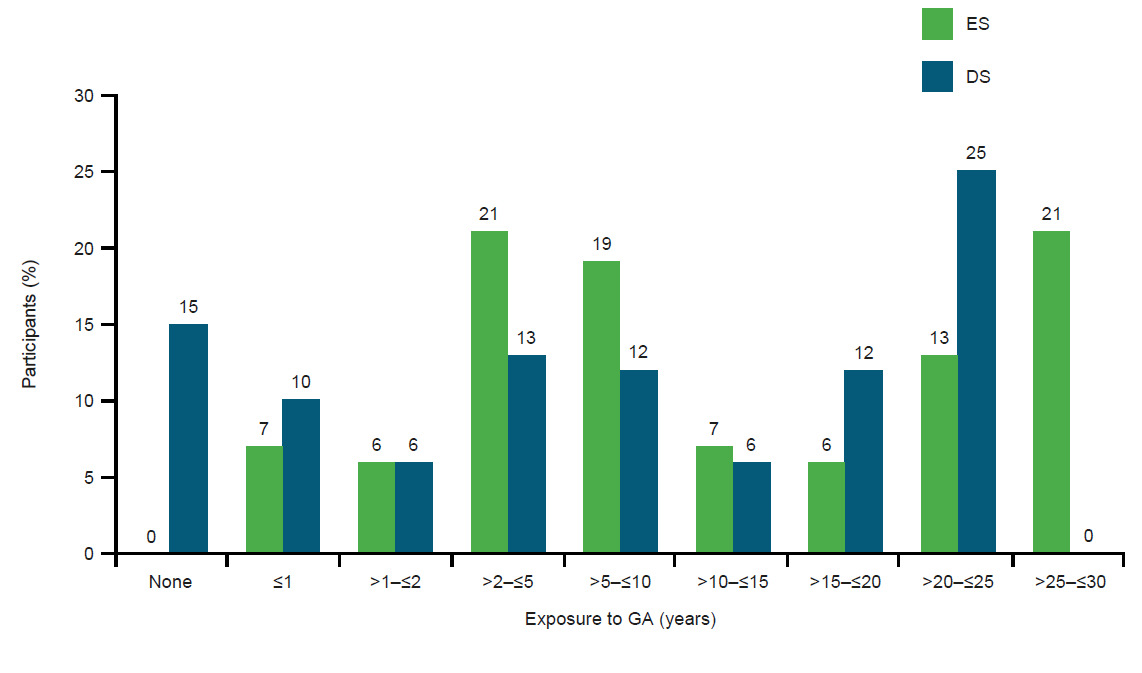


ES: early start; DS: delayed start; GA: glatiramer acetate.
